# The Efficacy and Safety of Probiotic Combinations Lobun Forte® Versus Renadyl® in Patients With Chronic Kidney Disease: A Comparative, Phase IV, Randomized, Open-Label, Active-Controlled, Parallel Study

**DOI:** 10.7759/cureus.67987

**Published:** 2024-08-28

**Authors:** Raja Karthik Kalidindi, C Prabhakar Reddy, Kishan PV, Prasad Kompella

**Affiliations:** 1 Nephrology, Nizam's Institute of Medical Sciences, Hyderabad, IND; 2 Clinical Pharmacology and Therapeutics, Nizam’s Institute of Medical Sciences, Hyderabad, IND; 3 Clinical Pharmacology, Sanzyme P Ltd, Hyderabad, IND; 4 Chemistry, Sanzyme P Ltd, Hyderabad, IND

**Keywords:** multi-strain probiotic supplement, malnutrition, uremic toxins, quality of life (qol), chronic kidney disease (ckd)

## Abstract

Background: Chronic kidney disease (CKD) often leads to gut microbiota imbalance, accelerating disease progression and increasing uremic toxins and inflammation. We conducted a randomized clinical trial comparing outcomes between two multi-strain probiotic supplements Lobun Forte® (Sanzyme P Ltd, Hyderabad, India) containing *Streptococcus thermophilus*, *Lactobacillus acidophilus*, *Bifidobacterium longum*, and *Bacillus coagulans* and Renadyl^®^ (Kibow Biotech, LLC., Pennsylvania, United States) containing *S. thermophilus*, *L. acidophilus,* and *B. longum*.

Materials and Methods: Sixty patients with stage 3-4 CKD were randomized to receive either Lobun Forte (n=30) or Renadyl (n=30) for six months, with each supplement providing 45 billion CFU/capsule, twice daily. Primary outcomes included quality of life (QoL) (Short-Form 8 (SF-8) score), reductions in uremic toxins (p-cresyl sulfate (PCS), 3-indoxyl sulfate (IS), indole-3-acetic acid (IAA)), blood urea nitrogen (BUN), serum creatinine, and serum uric acid. Secondary outcomes assessed oxidative stress, inflammatory biomarkers, and estimated glomerular filtration rate (eGFR).

*Results*: Both Lobun Forte and Renadyl groups showed significant improvements in QoL, with Lobun Forte achieving a 53.5% improvement (16.43 point increase) and Renadyl a 51.1% improvement (15.27 point increase) in SF-8 scores (p < 0.0001). The levels of IS decreased significantly in both groups (p < 0.0001), with Lobun Forte reducing IS by 29.72% and Renadyl by 24.20%. In terms of other uremic toxins, Lobun Forte showed non-significant (p > 0.05) reductions in mean PCS (7.63%) and IAA (15.57%), whereas Renadyl demonstrated a significant (p = 0.0314) decrease in PCS (20.75%) and a non-significant (p > 0.05) reduction in IAA (12.35%). Both groups showed significant (p < 0.0001) reductions in BUN and serum creatinine levels. Serum uric acid levels showed a significant (p = 0.0448) reduction with Lobun Forte while Renadyl exhibited a non-significant reduction (p = 0.1034).

Lobun Forte significantly (p = 0.0359) reduced mean high-sensitivity C-reactive protein (hsCRP) levels, while Renadyl showed a non-significant reduction (p = 0.0876). Both groups had non-significant reductions in interleukin-6 and tumor necrosis factor-alpha levels (p > 0.05). Further, both groups experienced significant (p < 0.0001) increases in mean glutathione levels and nitric oxide levels. Additionally, Renadyl resulted in a significant reduction in mean malondialdehyde, whereas Lobun Forte showed a non-significant reduction. Both probiotics significantly (p < 0.0001) improved eGFR, with Lobun Forte increasing it by 40.4% and Renadyl by 36.9%. Both probiotics were well tolerated, with a favorable safety profile throughout the study.

*Conclusion*: Both Lobun Forte and Renadyl effectively improve the quality of life in patients with stage 3-4 CKD by modulation of uremic toxins, renal parameters, inflammatory biomarkers, oxidative biomarkers, and eGFR. These findings suggest that both probiotics may help delay CKD progression by modulating the gut-kidney axis.

## Introduction

Chronic kidney disease (CKD) is a global public health problem that affects more than 10% of the adult population and is associated with increased morbidity and mortality [1]. It is characterized by progressive loss of renal function and accumulation of metabolic waste products such as urea, creatinine, and uric acid, in the blood. These waste products, also known as uremic toxins, can cause systemic inflammation, oxidative stress, endothelial dysfunction, and cardiovascular complications and are the leading causes of death in patients with CKD [2].

The gut microbiota, the complex community of microorganisms that inhabit the gastrointestinal tract, plays a crucial role in the production and metabolism of uremic toxins. In healthy individuals, the gut microbiota maintains a symbiotic relationship with the host, providing benefits such as nutrient digestion, vitamin synthesis, immune modulation, and protection against pathogens [3]. However, in patients with CKD, the gut microbiota undergoes significant changes, such as reduced diversity, increased abundance of pathogenic bacteria, and decreased abundance of beneficial bacteria. This dysbiosis leads to increased production of uremic toxins, such as indoxyl sulfate (IS), p-cresyl sulfate (PCS), and indole-3-acetic acid (IAA), which are derived from the bacterial fermentation of dietary protein and amino acids [4]. These toxins can cross the intestinal barrier and enter the systemic circulation, where they exert deleterious effects on various organs and tissues [5].

One of the potential strategies to modulate the gut microbiota and reduce the production and absorption of uremic toxins is the use of probiotics. Probiotics are defined as live microorganisms that, when administered in adequate amounts, confer a health benefit on the host [6]. Probiotics can influence the gut microbiota by competing with pathogenic bacteria, producing antimicrobial substances, enhancing the intestinal barrier function, and modulating the immune and inflammatory responses [7]. Probiotics support the microbiome and reduce inflammation and intestinal production of uremic toxins [8]. Several studies have suggested that probiotics may have beneficial effects in patients with CKD, such as improving the quality of life (QoL), reducing the levels of uremic toxins and inflammatory markers, and slowing the progression of renal impairment [9].

Despite the potential benefits of probiotics in patients with CKD, the existing evidence regarding their efficacy and safety remains limited and inconsistent. Most studies conducted thus far have been limited by small sample sizes and have used a variety of probiotic strains, doses, durations, and outcome measures. Moreover, the mechanisms by which probiotics exert their effects on the gut microbiota and the uremic toxins are not fully elucidated. Therefore, there is a need for more rigorous and comprehensive studies to evaluate the potential of probiotics as a therapeutic option for patients with CKD. In this study, we aimed to compare the efficacy and safety of Lobun Forte® (Sanzyme P Ltd, Hyderabad, India) and Renadyl® (Kibow Biotech, LLC., Pennsylvania, United States) in patients with CKD. Lobun Forte and Renadyl are two commercially available multi-strain probiotic combination products. Lobun Forte comprises *Streptococcus thermophilus*, *Lactobacillus acidophilus*, *Bifidobacterium longum*, and *Bacillus coagulans*, whereas Renadyl comprises *S. thermophilus*, *L. acidophilus*, and *B. longum*.

## Materials and methods

This study was conducted at the Department of Nephrology, Nizam's Institute of Medical Sciences (NIMS), Hyderabad, India. It was approved by the Institutional Ethics Committee, NIMS (approval number: EC/NIMS/2377/2019 dated August 24, 2019). The study was prospectively registered with the Clinical Trials Registry India (registration number: CTRI/2019/10/021546) and was conducted in accordance with good clinical practice (GCP), Declaration of Helsinki.

Study design

This phase IV study adopted an open-label, active-controlled, parallel design to compare the efficacy and safety of two probiotic multi-strain combinations, Lobun Forte and Renadyl, in patients with stages 3-4 CKD.

**Figure 1:**
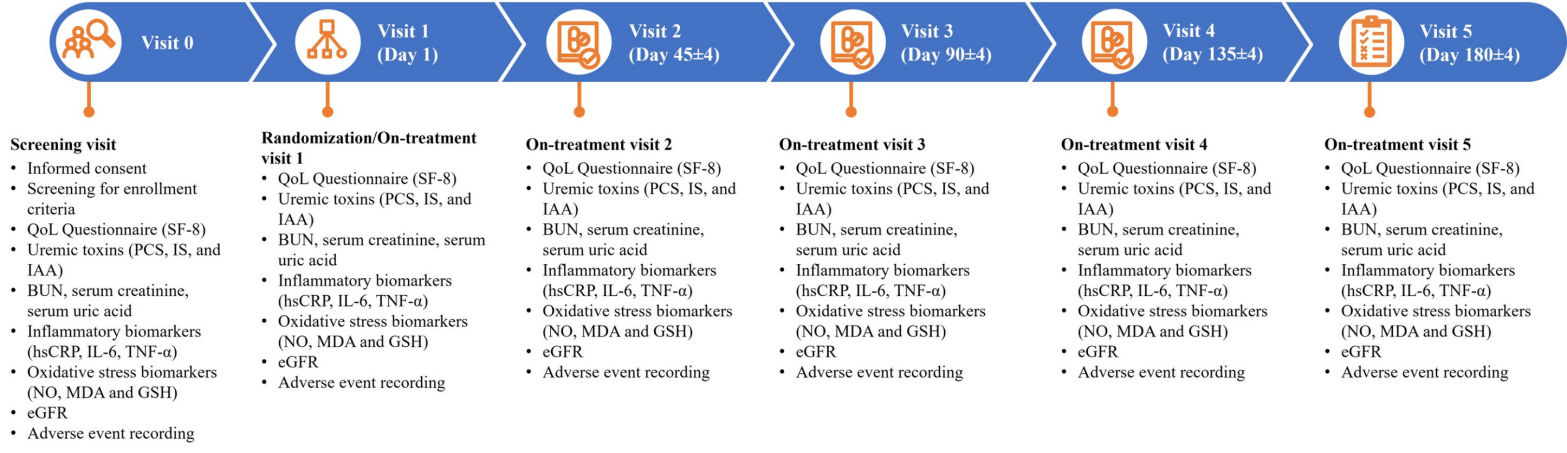
Study flow chart and respective evaluations at various study visits BUN: blood urea nitrogen; eGFR: estimated glomerular filtration rate; GSH: glutathione; hsCRP: high-sensitivity C-reactive protein; IAA: indole-3-acetic acid; IL-6: interleukin-6; IS: 3-indoxyl sulfate; MDA: malondialdehyde; NO: nitric oxide; PCS: p-cresol sulphate; QoL: quality of life; SF-8: short form-8; TNF-α: tumor necrosis factor-alpha. Image Credit: Raja Karthik Kalidindi and Kishan PV

Inclusion and exclusion criteria

Both male and female patients with CKD stages 3 or 4 who have been stable for at least six months and aged between 18 and 75 years, were enrolled. Stage 3 CKD was defined as a moderate reduction in glomerular filtration rate (GFR) at 30-59 mL/min/1.73 m^2^, and Stage 4 CKD was defined as a severe reduction in GFR (15-29 mL/min/1.73 m^2^); serum creatinine levels greater than 2.5 mg/dL at the time of screening.

Exclusion criteria encompassed pregnancy or lactation, antibiotic treatment within 14 days before screening, positive serologic tests for human immunodeficiency virus, hepatitis B surface antigen, or hepatitis C, active substance dependency, anticoagulant therapy, unwillingness to sign the informed consent form, administration of investigational drugs within 30 days of screening, active addictive drug or alcohol use, and any condition affecting safety or evaluation of study endpoints.

Study treatments

The study patients (N=60) were randomized in a 1:1 ratio into two groups: the Lobun Forte group (n=30) and the Renadyl group (n=30). Each patient received a respective multi-strain probiotic supplement containing 45 billion CFU per capsule twice daily for six months, as per the randomization schedule.

Outcome measures

The study involved five visits: screening visit (visit 0), randomization/baseline visit (visit 1 (day 0)), on-treatment visits (visit 2 (day 45±4), visit 3 (day 90±4), and visit 4 (day 135±4)), and end of study (EOS)/endpoint evaluation visit (visit 5 (day 180±4)). Study patients’ diaries were reviewed at each patient visit to the site.

At each visit, patients were assessed for primary outcome measures which included the improvement in QoL (measured using the Short-Form 8 (SF-8) score), reduction in levels of uremic toxins (PCS, IS, and IAA), blood urea nitrogen (BUN), serum creatinine, and serum uric acid.

Additionally, the patients were assessed for secondary outcome measures which include inflammatory biomarkers (high sensitivity C-reactive protein (hsCRP), interleukin-6 (IL-6), and tumor necrosis factor-alpha (TNF-α)), oxidative stress parameters (nitric oxide (NO), malondialdehyde (MDA), and glutathione (GSH)), and estimated GFR (eGFR) (Figure 1). Uremic toxins were measured using reversed-phase high-performance liquid chromatography [10]. Other parameters (hsCRP, IL-6, TNF-α, NO, MDA, GSH) were assessed using the enzyme-linked immunoassay (ELISA) technique [11].

Safety endpoints included recording adverse events (AEs) and severe AEs (SAEs), subject withdrawals due to toxicities, and changes from baseline in clinical laboratory values and vital signs. AEs were assessed at each visit and via additional phone calls. Safety parameters considered include incidence, severity, causality, and outcomes of AEs.

Statistical analysis

Descriptive statistics summarized safety data, and efficacy analysis employed the intention-to-treat population. Parametric or non-parametric tests were applied based on data distribution. Statistical methods included mean, and percentage change calculations. Paired *t*-test was done to compare the mean percentage changes in both the treatment groups before and after probiotic treatment (visit 1 and visit 5, respectively). Statistical analyses utilized GraphPad PRISM software (Insightful Science Holdings, LLC , San Diego, California, United States) with a significance level of p < 0.05.

## Results

All the randomized patients successfully completed the six-month study period, and there were no dropouts from the study. At the study's onset, there were no significant differences in age, body weight, or mean body mass index between the two groups (p > 0.05) (Table 1). Treatment adherence was robust, with all 60 patients (100%) in both the Lobun Forte (n=30) and Renadyl (n=30) groups consuming over 80% (more than 288 doses across 180 days) of their assigned medication. This underscores the acceptability of both Lobun Forte and Renadyl. 

**Table 1:** Baseline demographics A value of p < 0.05 is defined as statistically significant. SD: standard deviation.

Patient Characteristics	Lobun Forte^®^ group (n=30)	Renadyl^®^ group (n=30)	p-value
Age, in years, mean±SD	55.7±13.1	56.1±12.8	p = 0.9052
Gender: Males, n (%)	19 (63.3)	18 (60.0)	p = 0.8694
Body mass index (kg/m^2^), mean±SD	26.4±4.1	25.8±4.5	p = 0.5914

Primary outcome measures

All the primary outcome measures were shown as mean±SD for visit 1 and visit 5, and the mean percentage change between the visits and p values using paired t test is shown in Table 2.

**Table 2:** Effect of probiotic supplementations on various parameters in patients with CKD stages 3 and 4 A value of p < 0.05 is defined as statistically significant. The p values in bold text show statistical significance (p < 0.05). BUN: blood urea nitrogen; CKD: chronic kidney disease; eGFR: estimated glomerular filtration rate; GSH: glutathione; hsCRP: high sensitivity C-reactive protein; IAA: indole-3-acetic acid; IL-6: interleukin 6; IS: 3-indoxyl sulfate; MDA: malondialdehyde; NO: nitric oxide; PCS: p-cresol sulphate; QoL: quality of life; SD: standard deviation; SF-8: short form-8 health survey; TNF- α: tumor necrosis factor -alpha.

Parameters		Lobun Forte^®^ group (n=30)				Renadyl^®^ group (n=30)			
		Baseline (Visit 1), Mean±SD	Day 180 (Visit 5), Mean±SD	Mean difference, % (n)	p-value (Baseline vs Day 180)	Baseline (Visit 1), Mean±SD	Day 180 (Visit 5), Mean±SD	Mean difference % (n)	p-value (Baseline vs Day 180)
	SF-8 Score	14.30±5.09	30.73±4.34	53.5 (16.43)	<0.0001	14.60±5.61	29.87±2.82	51.1 (15.27)	<0.0001
QoL									
Uremic toxins	PCS (ng/mL)	14.03±5.15	12.96±6.51	7.6 (1.02)	0.9627	13.69±5.08	10.85±4.21	20.8 (2.84)	0.0314
	IS (ng/mL)	12.40±5.89	8.72±5.08	29.7 (3.68)	<0.0001	11.98±5.30	9.08±4.42	24.2 (2.90)	<0.0001
	IAA (ng/mL)	3.79±2.14	3.20±1.79	15.6 (0.59)	0.8198	3.52±2.24	3.09±1.16	12.4 (0.43)	0.7654
Renal markers	BUN (mg/dL)	80.83±11.44	61.93±10.72	23.4 (18.90)	<0.0001	80.63±7.83	61.47±7.06	23.8 (19.16)	<0.0001
	Serum creatinine (mg/dL)	3.57±1.24	3.12±1.12	12.8 (0.46)	0.0001	3.67±1	3.17±0.94	13.6 (0.50)	<0.0001
	Serum uric acid (mg/dL)	5.61±1.21	4.98±0.11	11.2 (0.63)	0.0448	6.42±1.39	5.93±0.74	7.6 (0.49)	0.1034
Inflammatory biomarkers	hsCRP (mg/L)	5.79±2.85	3.91±1.67	32.6 (1.90)	0.0359	5.29±2.28	3.98±2.05	24.8 (1.31)	0.0876
	IL-6 (pg/mL)	6.73±3.82	6.18±2.71	8.2 (0.55)	0.9464	6.43±2.14	5.38±1.36	16.4 (1.05)	0.2122
	TNF-α (pg/mL)	3.42±1.28	2.97±1.75	13.12 (0.45)	0.687	3.83±1.48	2.84±1.82	25.8 (0.99)	0.146
Oxidative stress markers	MDA (µmol)	9.06±7.04	7.81±3.75	13.8 (1.25)	0.9035	9.52±1.97	7.44±4.00	21.8 (2.08)	0.0318
	GSH (µmol)	381.0-±147.20	607.60±261.20	59.5 (226.60)	0.0001	404.20±85.03	582.20±134.70	44.0 (178)	<0.0001
	NO (µmol)	11.78±7.14	17.94±7.27	52.3 (6.16)	<0.0001	12.38±6.96	19.76±10.35	59.6 (7.38)	<0.0001
eGFR (ml/min)		32.02±12.95	44.96±12.99	40.4 (12.94)	<0.0001	32.14±12.88	44.01±12.81	36.9 (11.87)	<0.0001

Both Lobun Forte and Renadyl groups demonstrated significant improvements in QoL, as measured by SF-8 scores (p < 0.0001). The Lobun Forte group showed a 53.5% improvement (16.43 point increase), while the Renadyl group exhibited a 51.1% improvement (15.27 point increase). The levels of IS decreased significantly in both groups (p < 0.0001). Lobun Forte administration resulted in a mean reduction of 29.7% (3.68 ng/mL), whereas Renadyl treatment led to a 24.2% decrease (2.90 ng/mL).

Regarding other uremic toxins, the Lobun Forte group experienced non-significant reductions (p > 0.05) in mean PCS levels by 7.6% (1.02 ng/mL) and IAA levels by 15.6% (0.59 ng/mL). In contrast, the Renadyl group showed a significant decrease (p < 0.05) in mean PCS levels by 20.8% (2.84 ng/mL), but a non-significant reduction (p > 0.05) in IAA levels by 12.4% (0.43 ng/mL).

Both groups exhibited significant improvements in kidney function markers. BUN decreased by 23.4% (18.9 mg/dL, p < 0.0001) in the Lobun Forte group and by 23.8% (19.16 mg/dL, p < 0.0001) in the Renadyl group. Serum creatinine levels were reduced by 12.8% (0.46 mg/dL, p = 0.0001) in the Lobun Forte group and by 13.6% (0.50 mg/dL, p = 0.0001) in the Renadyl group. Serum uric acid levels showed a significant 11.2% reduction (0.63 mg/dL, p = 0.0448) in the Lobun Forte group, while the Renadyl group experienced a non-significant 7.6% reduction (0.49 mg/dL, p = 0.1034).

Secondary outcome measures

All the secondary outcome measures were shown as mean±SD for visit 1 and visit 5, and the mean percentage change between the visits and p value using paired t test is shown in Table 2.

The Lobun Forte group exhibited a significant reduction in mean hsCRP levels by 32.56% (1.90 mg/L) (p = 0.0359), while the Renadyl group showed a non-significant reduction of 24.78% (1.31 mg/L) (p = 0.0876). Both groups demonstrated non-significant reductions in IL-6 and TNF-α levels (p > 0.05).

Regarding oxidative stress parameters, the Lobun Forte group displayed a 59.5% (226.6 µmol) increase in mean GSH levels (p = 0.0001) and a 52.3% (6.16 µmol) increase in mean NO levels (p < 0.0001). Similarly, the Renadyl group exhibited a 44.0% (178 µmol) increase in mean GSH levels (p < 0.0001) and a 59.6% (7.38 µmol) increase in mean NO levels (p < 0.0001). Additionally, the Renadyl group showed a significant reduction in mean MDA levels by 21.8% (2.08 µmol) (p = 0.0318), whereas the Lobun Forte group demonstrated a non-significant reduction of 13.8% (1.25 µmol) (p = 0.9035).

Moreover, both groups (Lobun Forte and Renadyl) displayed significant improvements in eGFR; Lobun Forte with a 40.4% (12.94 ml/min) increase (p < 0.0001) and Renadyl with a 36.9% (11.87 ml/min) increase (p < 0.0001).

**Figure 2:**
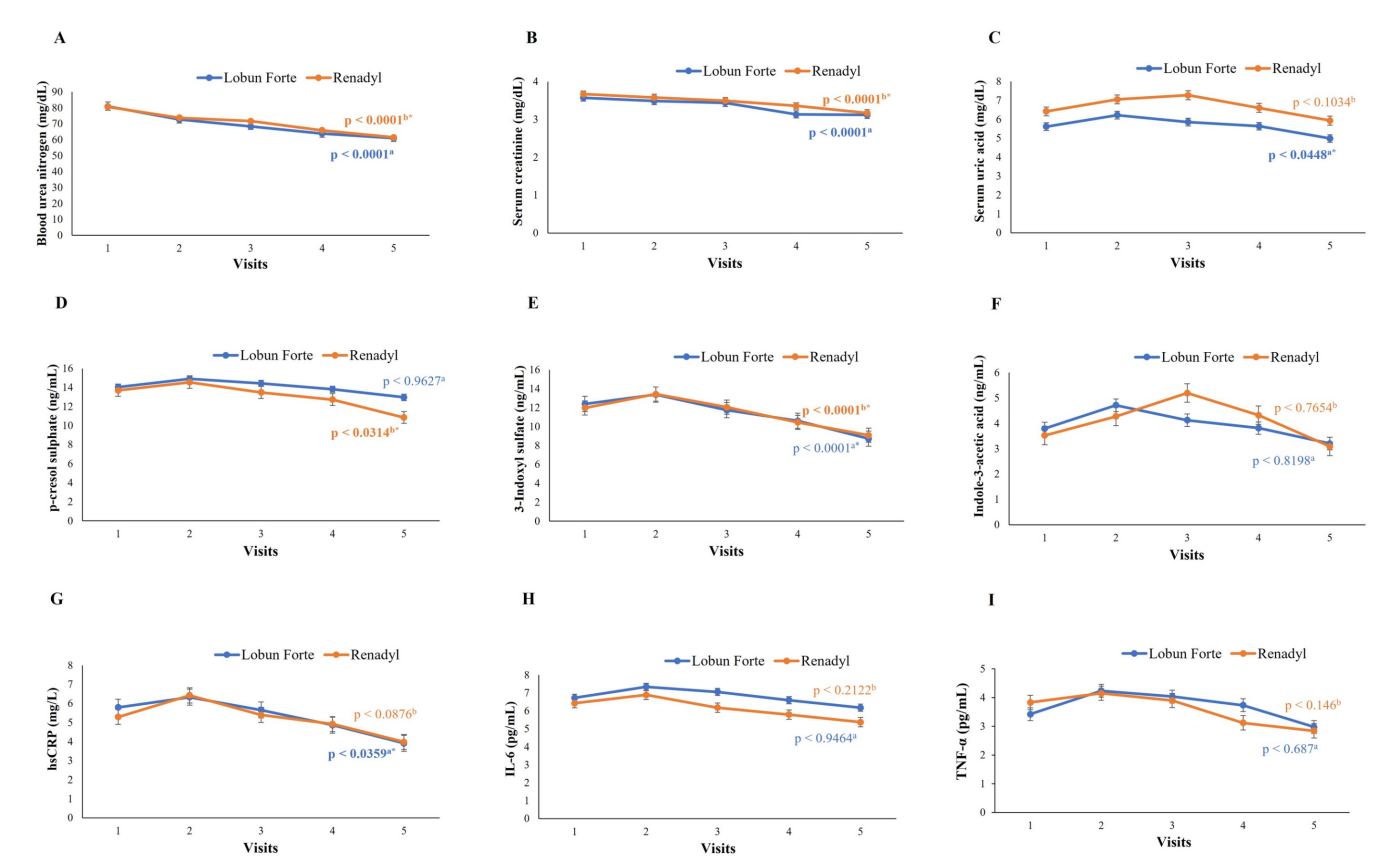
Trajectory of change from baseline to day 180 in various assessments, including renal parameters (A-C), uremic toxins (D-F), and inflammatory biomarkers (G-I) *A value of p < 0.05 is defined as statistically significant. ^a^Lobun Forte® group Baseline vs Day 180; ^b^Renadyl® group Baseline vs Day 180. (A) Blood urea nitrogen and (B) serum creatinine decreased significantly in both groups (p < 0.0001). (C) Serum uric acid decreased significantly with Lobun Forte® (p < 0.0448). (D) p-cresol sulphate and (E) 3-Indoxyl sulfate reduced significantly with Renadyl® (p < 0.0314 and p < 0.0312, respectively). (F) indole-3-acetic acid showed no significant changes. (G) hsCRP decreased significantly with Lobun Forte® (p < 0.0359). (H) IL-6 and I) TNF-α showed no significant changes in either group. hsCRP: high-sensitivity C-reactive protein; IL-6: interleukin-6; TNF-α: tumor necrosis factor-alpha

The trajectories of the evaluations from baseline to day 180 are presented in Figure 2. These findings suggest that both probiotic combinations are effective in managing CKD, with Lobun Forte potentially offering benefits in additional parameters.

Overall, oral administration of Lobun Forte and Renadyl was well tolerated and demonstrated a favorable safety profile throughout the trial period.

## Discussion

Previous research reports and the draft Kidney-Disease: Improving Global Outcomes (KDIGO) guidelines suggest that probiotics support the microbiome and reduce inflammation and intestinal production of uremic toxins [8,12]. In the current study, we compared the efficacy and safety of two multi-strain probiotic combinations, Lobun Forte consisting of *S. thermophilus*, *L. acidophilus*, *B. longum*, and *B. coagulans*, and the Renadyl group comprising *S. thermophilus, L. acidophilus, and B. longum*, in patients with stages 3 and 4 CKD. We hypothesized that Lobun Forte and Renadyl would enhance QoL and decrease uremic toxins and inflammatory markers in patients with CKD. We anticipated Lobun Forte to be more effective due to its spore-forming probiotics (*B. coagulans*).

CKD is associated with dysbiosis of the gut microbiota, which leads to increased production and absorption of uremic toxins, such as PCS, IS, and IAA [5,9]. These toxins contribute to the progression of kidney damage and cardiovascular complications [5,9,13]. Probiotics can modulate the gut microbiota and reduce the production and absorption of uremic toxins by competing with pathogenic bacteria, producing short-chain fatty acids, and enhancing the intestinal barrier [14-16]. In this study, both the Lobun Forte group and the Renadyl group showed a decreasing trend in PCS, IS, and IAA levels. This aligns with the known benefits of probiotics in CKD, including the reduction of uremic toxins [17,18].

Inflammation is another key factor in the pathogenesis and outcomes of CKD [19]. Elevated levels of inflammatory cytokines, such as C-reactive protein (CRP), IL-6, and TNF-α, are associated with increased mortality, cardiovascular events, and protein-energy wasting in patients with CKD [20-22]. It is evident from several previous studies that probiotics can modulate the immune system and reduce inflammation by influencing the gut-associated lymphoid tissue, producing anti-inflammatory substances, and regulating the expression of inflammatory genes [23]. In alignment with the previous studies, in this study, the Lobun Forte and Renadyl groups both showed a decreasing trend in CRP, IL-6, and TNF-α levels, with a significant reduction in CRP in the Lobun Forte group and a significant reduction in MDA in the Renadyl group.

Oxidative stress, characterized by an imbalance between oxidants and antioxidants, is a common feature in CKD [19]. Probiotics can reduce oxidative stress by enhancing the antioxidant defense system, scavenging free radicals, and inhibiting the activation of oxidative pathways [24]. In our study, significant improvements in oxidative stress biomarkers (GSH and NO) were observed in both treatment groups. This suggests that Lobun Forte and Renadyl probiotic combinations can help mitigate oxidative stress in patients with CKD, which is consistent with existing literature [24].

The goal of probiotic therapy in patients with CKD is to preserve or improve kidney function and delay the need for dialysis or transplantation [25]. In this study, both probiotic combinations showed significant reductions in BUN and serum creatinine, which are indicators of kidney function. Moreover, both probiotic combinations showed significant improvements in eGFR, which is a more accurate measure of kidney function. These results suggest that probiotics can slow down the progression of CKD by reducing the uremic burden, inflammation, and oxidative stress. However, the clinical significance of these changes needs to be further evaluated in long-term studies.

Another important aspect of probiotic therapy in patients with CKD is the impact on QoL, which is often impaired by the symptoms and complications of the disease [26,27]. Probiotics can improve QoL by alleviating gastrointestinal symptoms such as constipation, diarrhea, bloating, and abdominal pain, which are common in patients with CKD [28]. Probiotics can also improve psychological well-being, such as mood, anxiety, and depression, by modulating the gut-brain axis. In this study, both the Lobun Forte and Renadyl groups improved QoL as reflected in SF-8 scores which measure both physical and mental health. These results indicate that probiotics can improve the subjective experience and satisfaction in patients with CKD. This study underscores the holistic benefits of both probiotics, making them promising options for patients with CKD. To our knowledge, this is the first study comparing the efficacy and safety of two probiotic combinations in patients with stage 3-4 CKD.

The strengths of this study include its randomized, open-label, active-controlled, parallel design, high medication adherence rate, and comprehensive assessment including QoL. However, the study limitations include a small sample size, short duration of treatment, and lack of a placebo group. The results should be interpreted with caution and need confirmation from larger, longer-term studies with a placebo-controlled design and standardized probiotic formulations.

## Conclusions

The results of this study provide strong evidence for the comparable efficacy of Lobun Forte with Renadyl in managing key parameters in patients with stage 3-4 CKD and thereby delay the progression of CKD. The unique profile of benefits demonstrated by Lobun Forte, including its potential anti-inflammatory benefits and superior efficacy in modulating uremic toxins, renal parameters, oxidative stress markers, and e-GFR, make it a promising therapeutic option for patients with CKD. The robust treatment adherence and improvement in QoL further underscore the potential role of Lobun Forte in delaying the progression of CKD. These findings warrant further investigation in larger multi-center trials to confirm the benefits of Lobun Forte in a broader CKD population.

## Appendices

SF 8 questionnaire

1. Overall, how would you rate your health during the past 4 weeks?

Excellent, Very good, Good, Fair, Poor, Very poor

2. During the past 4 weeks, how much did physical health problems limit your usual physical activities (such as walking or climbing stairs)?

Not at all, Very little, Somewhat, Quite a lot, Could not do physical activities

3. During the past 4 weeks, how much difficulty did you have doing your daily work, both at home and away from home, because of your physical health?

None at all, A little bit, Some, Quite a lot, Could not do daily work

4. How much bodily pain have you had during the past 4 weeks?

None, Very mild, Mild, Moderate, Severe, Very Severe

5. During the past 4 weeks, how much energy did you have?

Very much, Quite a lot, Some, A little, None

6. During the past 4 weeks, how much did your physical health or emotional problems limit your usual social activities with family or friends?

Not at all, Very little, Somewhat, Quite a lot, Could not do social activities 7. During the past 4 weeks, how much have you been bothered by emotional problems (such as feeling anxious, depressed, or irritable)?

Not at all, Slightly, Moderately, Quite a lot, Extremely

8. During the past 4 weeks, how much did personal or emotional problems keep you from doing your usual work, school, or other daily activities?

Not at all, Very little, Somewhat, Quite a lot, Could not do daily activities
